# Treatment of a Patient with Borderline Personality Disorder Based on Phase-Oriented Model of Eye Movement Desensitization and Reprocessing (EMDR): A Case Report

**Published:** 2018-01

**Authors:** Nahid Momeni Safarabad, Ali-Asghar Asgharnejad Farid, Banafsheh Gharraee, Mojtaba Habibi

**Affiliations:** 1 Department of Clinical Psychology, Tehran Institute of Psychiatry, School of Behavioral Sciences and Mental Health, Iran University of Medical Sciences, Tehran, Iran.; 2 Department of Health Psychology, School of Behavioral Sciences and Mental Health (Tehran Institute of Psychiatry), Iran University of Medical Sciences, Tehran, Iran.

**Keywords:** *Borderline Personality Disorder*, *Dissociative Symptoms*, *Eye Movement Desensitization and Reprocessing*

## Abstract

**Objective:** This study aimed at reporting the effect of the 3-phase model of eye movement desensitization and reprocessing in the treatment of a patient with borderline personality disorder.

**Method** **:** A 33-year-old female, who met the DSM-IV-TR criteria for borderline personality disorder, received a 20-session therapy based on the 3-phase model of eye movement desensitization and reprocessing. Borderline Personality Disorder Checklist (BPD-Checklist), Dissociative Experience Scale (DES-II), Beck Depression Inventory-II-second edition (BDI-II), and Anxiety Inventory (BAI) were filled out by the patient at all treatment phases and at the 3- month follow- up.

**Results:** According to the obtained results, the patient’s pretest scores in all research tools were 161, 44, 37, and 38 for BPD-Checklist, DES-II, BDI-II, and BAI, respectively. After treatment, these scores decreased significantly (69, 14, 6 and 10 respectively). So, the patient exhibited improvement in borderline personality disorder, dissociative, depression and anxiety symptoms, which were maintained after the 3-month follow-up.

**Conclusion: **The results supported the positive effect of phasic model of eye movement desensitization and reprocessing on borderline personality disorder.

Borderline personality disorder is a disempowering condition, which is associated with primary life stressors (1). Trauma experts maintain that BPD might be a complex variation of PTSD (2). Presence of severe dissociation symptoms in these patients might prolong the treatment (3). Thus, it is suggested that such patients require a trauma-focused treatment, such as dialectic behavior therapy for PTSD (DBT-PTSD) and cognitive-behavior therapy for PTSD (CBT-PTSD). Although studies have shown that DBT-PTSD is a supported approach for BPD comorbid with PTSD, it does not directly target PTSD.

 Furthermore, it has been found that treatment approaches, such as cognitive-behavior therapy (prolonged exposure), that focus primarily on the processing of traumatic memories are not appropriate for chronic traumatic individuals (4). Thus, trauma experts consent to integrate their therapeutic approach within a phase-oriented model for BPD comorbid with PTSD and high level of dissociation. Therefore, phase-oriented model of eye movement desensitization and reprocessing has been suggested for these patients (5-7). 

Eye movement desensitization and reprocessing, designed by Shapiro, is a trauma-focused method of therapy, whose effectiveness in reduction of PTSD symptoms in controlled studies has been proved (8). Despite the significant relation between PTSD and BPD, only few studies have been published on the application of eye movement desensitization and reprocessing method in the treatment of patients suffering from BPD and its comorbid disorders (7). 

For instance, a case study conducted on 2 female participants revealed the effectiveness of 3 sessions of eye movement desensitization and reprocessing therapy based on resource development and installation (RDI) protocol in the reduction of some BPD symptoms (anger, self-destructive behavior, and binge eating), anxiety, depression, as well as trauma symptoms. The results of the therapy maintained after a one-month follow-up (9). Another study on a female patient revealed the beneficial effects of 20 sessions of eye movement desensitization and reprocessing therapy, which was based on the standard protocol in reduction of BPD symptoms. The positive effects were maintained in this study after a 7-month follow-up (10). According to these preliminary studies, which were done based on the standard protocol of eye movement desensitization and reprocessing and the first phase of phasic model of eye movement desensitization and reprocessing, researchers supposed that treatment of BPD patients, who also have a history of trauma along with dissociative disorder or symptoms, requires a change in the standard protocol of eye movement desensitization and reprocessing. They also maintained that treatment should be focused on increasing resources, identifying and working on dissociative phobias, and targeting trauma symptoms; and they noted that reconstructing personality and the efficacy of this new model need research (7). Hence, the present study aimed at evaluating the effectiveness of phasic model of eye movement desensitization and reprocessing in reduction of BPD symptoms in a female patient with history of chronic trauma and high level of dissociation. 

*Patient Information, Therapeutic Interventions and Outcomes*

The patient was a 33-year-old, single, and unemployed female with a BA degree in Social Sciences. She was diagnosed with BPD comorbid with major depression disorder by several psychiatrists, based on DSM-IV-TR criteria, and referred to Sedigh Clinic in Khorramabad, Lorestan, for psychotherapy. The patient was evaluated by administering the SCID-I and SCID-II for Axis I and Axis II disorders. She had no history of hospitalization or psychotherapy. Her borderline symptoms started in her teenage years. Severe mood swings along with periods of depression and emptiness had caused a dramatic drop in her performance at individual and social levels. She had experienced recurring anger attacks, which were mainly directed towards family members. She threatened to commit suicide every time and one month before the study, she attempted suicide by taking 25 diazepam 5mg pills. She was taken to the emergency department of a hospital and her stomach was washed; she was discharged after 2 days of hospitalization. Then, her family took her to a psychiatrist and he ordered doxepin 25 mg, citalopram 20 mg, and clonazepam 1mg and the dose was fixed throughout the study. She complained about recurring periods of binge eating, prodigality, lack of control over her emotions, extreme depression, derealization, depersonalization, and recurring periods of amnesia. She reported that she was sexually abused at the age of 8 by one of her teachers, which brought about a significant drop in her educational performance as well as symptoms of anxiety and severe panic. She did not tell anyone about it. She was also abused during her teenage years by one of her boyfriends. Before treatment, she gave verbal consent to participate in the study. Then, she received 20 weekly sessions of eye movement desensitization and reprocessing for 5 months. Treatment had 3 phases: (1) resource development and installation (RDI) phase, (2) trauma processing phase, and (3) personality rehabilitation phase. The aim of the first phase was to develop skills in the patient to cope with dissociative and self-injurious behaviors; the second phase helped the patient desensitize the traumatic memories; and, the third phase targeted future worries. The treatment was done by the first author of the present study (a PhD student of clinical psychology). The first 4 sessions were dedicated to the resource development and installation (RDI) phase, 12 sessions to the desensitization phase, and the last 4 sessions to personality rehabilitation phase. The Iranian version of BPD-checklist, DES-II, BDI-II, and BAI were completed by the patient at pretreatment, at the end of each treatment phase, and at 3-month follow-up. All measures have good psychometric properties. For instance, the internal consistency of BPD-checklist is high and split-half reliability coefficient of the Persian version of this checklist is 0.82. The test-retest reliability of the Persian version of DES-II is high as well (0.96). Furthermore, BDI-II and BAI has good validity and reliability (11-14). In the first session of resource development and installation phase, the patient’s required resources as well as their images were identified, and 2 resources were installed in each session. The patient exhibited her tendency to increase her capacity for self-acceptance, acquire the capability to soothe herself, increase her trust in the treatment, have more control over her emotions, be happier, and improve her skills for creating healthier relationships. Positive memories and images were identified for each of these resources and were installed using eye movements. After resources were improved and after the therapist confirmed that the patient was prepared to work on her traumas, the 8-step standard protocol of eye movement desensitization and reprocessing was presented to her. The patient initially showed extreme fear of focusing on her traumatic experiences, thus her fears were targeted by therapy. After the full processing of her traumatic experiences, the final phase of the treatment was conducted. At this stage, the patient’s current concerns and her future fears associated with finding a job, education, independence from family, finding a partner, as well as her feelings of the treatment were targeted. The obtained results revealed a significant decrease in BPD-checklist, BDI-II, and BAI inventory scores since the first phase of the treatment, which was continued up to the termination of treatment period. The results pertaining to DES-II scale showed that the changes in dissociation symptoms started to take place after the second phase of the treatment, and the patient’s score of this scale decreased significantly up to the end of the treatment. At the end of treatment, she did not have DSM-IV-TR criteria for borderline personality disorder and major depression. The positive effects of the treatment were maintained at 3-month follow-up (Figure 1).

**Figure 1:**
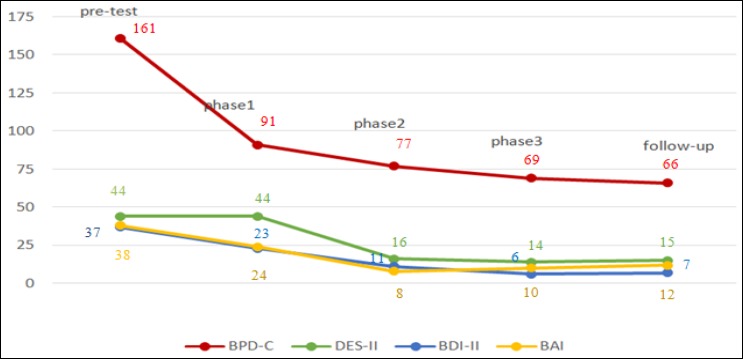
Improvement in Dependent Variables during Treatment Phases

Note: BPD-C: Borderline Personality Disorder Checklist; DES: Dissociative Experience Scale; BDI: Beck Depression Inventory; BAI: Beck Anxiety Inventory; Pre-t: Pretreatment; Phase 1: Resource Development Installation Phase; Phase 2: Trauma Processing Phase; Phase 3: Personality Reintegration and Rehabilitation Phase; Fu-up: Follow- up

## Discussion

The results of the present study indicated the positive effect of phasic model of eye movement desensitization and reprocessing on the treatment of BPD as well as improvement of dissociation, anxiety, and depression symptoms. Results of this study were consistent with prior findings supporting the efficacy of eye movement desensitization and reprocessing in improving BPD symptoms (9, 10). A dramatic decrease in the BPD, anxiety, and depression symptoms immediately after the resource development and installation phase, which conformed to a previous study conducted by Korn and Leads, (2002) showed the significance of this stage of treatment as an equipping or ego-strengthening skill-oriented intervention for decreasing BPD symptoms and negative emotions (9). Desensitization and reprocessing of early traumatic memories with eye movement desensitization and reprocessing helped the patient increase her access to adaptive information related to distressing memories. It also appeared that the processing of traumatic memories caused the patient to develop healthier beliefs, emotions, and behaviors (10). Additionally, targeting current problems and future concerns helped the patient develop more flexible and appropriate solutions and increase her capacity to face future challenges (6). Finally, duration of treatment is an important factor in the treatment of personality disorder (15). It seems that 5-month treatment in our study helped the patient to improve her mental condition and practice the gains of treatment in the real world. 

## Limitation

Even though the present study was the first one evaluating the effects of phasic model of eye movement desensitization and reprocessing on improving BPD, dissociation, anxiety, and depression symptoms through comparing the effects of each phase, the results should be interpreted discreetly as the study was done only on one patient, who may not represent the whole population of BPD patients. Additionally, one therapist delivered the treatment program. Thus, the characteristic of therapists rather than the treatment may affect the results. Finally, extended follow- up interval (12 month or longer) are needed to evaluate the sustainability of treatment gains, as duration of follow- up in the present study was short.

## Conclusion

Although the results of the present study are promising for BPD patients, more research should be done with larger sample sizes and with experimental and control groups to evaluate the positive effects of eye movement desensitization and reprocessing on BPD patients, also, more studies should be conducted to compare this phasic model of treatment with other phase-oriented treatments, such as DBT, CBT, and psychodynamic.
